# Antioxidant Activity in Frozen Plant Foods: Effect of Cryoprotectants, Freezing Process and Frozen Storage

**DOI:** 10.3390/foods9121886

**Published:** 2020-12-17

**Authors:** Lilia Neri, Marco Faieta, Carla Di Mattia, Giampiero Sacchetti, Dino Mastrocola, Paola Pittia

**Affiliations:** Faculty of Bioscience and Technologies for Food, Agriculture, and Environment, University of Teramo, Via Renato Balzarini 1, 64100 Teramo, Italy; lneri@unite.it (L.N.); mfaieta@unite.it (M.F.); cdimattia@unite.it (C.D.M.); gsacchetti@unite.it (G.S.); dmastrocola@unite.it (D.M.)

**Keywords:** fruits, vegetables, freezing, frozen storage, cryoprotectants, bioactive compounds, antioxidant activity

## Abstract

The antioxidant activity (AOA) of plant foods is recognized as an index of the potential health benefits resulting from their consumption. Due to their high perishability and seasonality, plant foods are largely consumed or used as processed products and freezing is one of the technologies used for the production of high-quality foods. However, cell breakages occurring during freezing and frozen storage can lead to the release of antioxidant compounds and their degradation due to chemical and enzymatic oxidation reactions, and thus, they could present a lower antioxidant activity compared to the corresponding fresh product. In this context, process conditions, freezing pre-treatments and the use of cryoprotectants can limit the extent of freeze-induced damages and preserve the antioxidant activity of plant foods. This review collects and discusses the state-of-the-art knowledge on the single and combined effect of freezing and frozen storage conditions on the antioxidant activity of fruits and vegetables as well as the role of cryoprotectants. Classes of compounds responsible for the antioxidant activity of plant foods and the most common methods used for the evaluation of the antioxidant activity in vitro are also presented. The freezing principles and the effects of ice nucleation and crystallization on fruits, vegetables and their main derivatives (juices, pulps) have been addressed to highlight their impact on the AOA of plant foods. The effect of freezing and frozen storage on the AOA of plant foods resulted dependant on a series of intrinsic factors (e.g., composition and structure), while the role of extrinsic processing-related factors, such as freezing and storage temperatures, is ambiguous. In particular, many conflicting results are reported in the literature with a high variability depending on the method of analysis used for the AOA evaluation and data expression (fresh or dry weight). Other intrinsic raw material properties (e.g., cultivar, ripening degree), post-harvest conditions, as well as defrosting methods that in the majority of the studies are scarcely reported, contribute to the aforementioned discrepancies. Finally, due to the limited number of studies reported in the literature and the high variability in product processing, the effect of cryoprotectants on the AOA of plant foods remains unclear.

## 1. Introduction

The daily consumption of plant foods is inversely related to the risk associated with the onset of many oxidative stress disorders [1,2,3,4,5,6]. The beneficial effects of plant consumption are attributed to the presence of bioactive molecules, including antioxidants [7,8,9]. The intake of antioxidants through food is of extreme importance since clinical trials using food antioxidants as dietary supplements have shown extremely contrasting results [10,11,12,13]. A possible explanation is the presence in foods of other components having higher activity than the well-known nutrient antioxidants. In particular, the beneficial effect of plant consumption seems to derive from the wide variety of bioactive compounds present in them and their interaction in vivo [7,14,15]. Furthermore, in most foods, several antioxidants are present and the total antioxidant activity results from the integrated and synergistic action of different compounds instead of the sum of every single compound [16].

Experimental evidence also suggests that antioxidants such as polyphenols undergo a complex metabolism process after ingestion, leading to the formation of bioactive metabolites and catabolic products of the human gut microbiota (HGM) and microbial enzymes activities. Hence, most biological properties of polyphenol-rich fruits and vegetables are attributed to their gut metabolites (GM) rather than their parent compounds [17].

The presence and concentration of antioxidant secondary metabolites in plant foods is due to numerous factors, including genetics, growing conditions (source availability, soil quality, insect and animal herbivory pressures) [18], ripening and post-harvest conditions [19]. Processing is another important factor influencing the antioxidant activity of plant foods. In fact, due to their high perishability and seasonality, these products are consumed or used not exclusively as fresh but also as processed products or in complex, and possibly processed, food formulations. Different preservation techniques can be exploited to ensure the quality, safety and shelf-life extension of plant foods. Among these, freezing is recognized as one of the main processes for long-term preservation and storage of fruits and vegetables and it is highly accepted by consumers who retain it to have a low impact on the nutritional quality of food products [20,21].

Freezing combines the preserving effect of low temperatures with the immobilization by crystallization of water in the form of ice to an extent that it is not available either as a solvent or reactive component. The decrease of temperature below the freezing point of the product allows the inhibition of metabolic processes occurring after harvesting and slows down the kinetics of microbiological growth and qualitative degradation reactions [22,23,24].

The limiting effect of freezing on chemical reactions is due to the reduction of molecular mobility and, thus, to (i) the reduction of the kinetic energy of the reagents, (ii) the subtraction of water from the system through crystallization, (iii) the increase in the viscosity of the unfrozen (non-crystalline) phase of the food with limiting effect on molecular mobility regardless of the thermodynamic state of the system and (iv) the crystallization of amorphous solutes with alteration of the composition of the concentrated phase.

While the low temperatures used in freezing processes may decrease the kinetic energy of the reagents, they may have controversial effects on oxidation reactions since both the increase in oxygen solubility at low temperatures, the concentration of the reagents in the non-frozen phase of the system and the crystallization of amorphous solutes may favor chemical and enzymatic oxidation reactions [25,26].

However, depending on the size and location of the ice crystals, freezing may damage cell membranes and break down the physical structure, and therefore the final quality of the product upon thawing may be lower than the corresponding fresh product. Moreover, freezing pre-treatments, conventionally applied to preserve the quality of frozen products by enzymatic inactivation, may impair the cell structure, and as a result, quality degradation including color changes, drip loss, softening and loss of nutrient and bioactive compounds are unavoidable [27,28].

On the contrary, some authors [29,30,31] have shown that freezing operations may also exert positive effects on the quality and functional properties of plant foods since the frozen state can favor the release of bioactive compounds as bound phenolic acids and anthocyanins, resulting in an increased antioxidant activity.

A common approach to limit the extent of freezing-induced damages and to efficiently preserve frozen food matrices also at industry level, is the use of cryoprotectants. This category includes molecules either naturally present or synthesized at need by vegetable matrices, which can be added to food formulations, hindering the loss of quality induced by freezing and cold storage [32,33]. Mechanisms of cryoprotection vary based on the nature of the molecule and its physical, chemical and physio-chemical properties. Depending on the type of mechanism exerted, these compounds can be classified in colligative or non-colligative cryoprotectants [34,35]. Their application can exert an effect on textural, rheological, nutritional, technological and sensorial properties of foods. However, their use has to comply with current food regulations, which in some cases can limit their use [36,37].

This paper aims to review and discuss the influence of freezing and frozen storage on the antioxidant activity of fruits and vegetables. After an introductory part on the classes of compounds responsible for the antioxidant activity of plant foods, the most common methods used for the evaluation of the in vitro antioxidant activity and the impact of the freezing and frozen storage on the physical properties and quality of frozen products, the review will extensively present and discuss the single and the combined effect of freezing and frozen storage on the antioxidant activity of both vegetables and fruits and their main derivatives (e.g., juices or pulps). Cryoprotectants, and their role in the preservation of the antioxidant activity of plant foods, will also be presented.

## 2. Freezing and Its Effect on Plant Foods

Food freezing is a thermodynamic process involving heat and mass transfer that consists in cooling the product to its freezing point, removing the latent heat of crystallization and further decreasing the temperature to the final storage temperature. Besides thermodynamic factors, which define the characteristics of the system under equilibrium conditions, it also involves kinetic ones that describe the rates at which equilibrium might be approached as water is converted into ice. Water crystallization is the key step determining both the efficiency of the process and the quality of frozen foods.

However, in plant food, both the technological parameters applied during freezing, the frozen state and other factors, including the initial raw material properties, post-harvest conditions and freezing pre-treatments, are relevant for maintaining the high qualitative attributes of the frozen product at thawing [22,38].

The phase transition of water to ice is a phenomenon consisting of two stages, i.e., the nucleation and the crystal growth [39]. The former implies the formation of a new crystal and occurs either in a crystal-free solution (primary nucleation) or in the presence of formerly created crystals (secondary nucleation) by supercooling the systems. Once formed, the growth of the ice crystals depends on the rate of removal of the latent heat released during the water phase change and the mass transfer of both water molecules from the surrounding solution and solutes (e.g., salts or sugar molecules) removed from the ice crystal surface [40].

Crystal nucleation and growth affect the crystal size distribution [41,42]. The number and size of ice formed in a frozen system depend on the initial supercooling (ΔTs = Tf − Ti, where Tf = final temperature and Ti = initial temperature), that affects both nucleation and growth rate. High supercooling values and freezing rates determine the formation of a large number of nuclei and ice is distributed in many small crystals, while low initial supercooling and freezing rates favor crystal growth, and thus, large crystals.

In food plant tissues, the cellular compartmentalization leads to the crystallization of local freezable water both at the intracellular and extracellular level, and due to the difference of osmotic pressure, this could generate the migration of unfrozen water to the frozen area in the cell and cell organelles. In particular, in cell plant foods, water moves from inside the vacuole through the tonoplast into the cytoplasm and then into the intercellular space across the cell membranes and cell wall [43]. Under these conditions, the liquid water is pulled out of the supercooled cells by determining the dehydration of the tissue (known as freeze concentration) as well as cell separation in the middle lamella region, causing cell wall rupture, cell shrinkage and collapse of the cell wall [44]. The cellular membranes lose their osmotic status and their semi-permeability [45].

The occurrence and entity of these phenomena depend on the freezing rate, freezing/thawing cycles, temperature variation during frozen storage, postharvest modifications and freezing pre-treatments, but also on the initial raw material properties with main reference to the structural properties of the plant tissue and cell membrane permeability [22]. The loss of water from the intracellular compartment could then affect turgidity, increase the intracellular solute concentration as well as the related physio-chemical (e.g., ionic strength, pH) and physical properties (e.g., viscosity) within the unfrozen portions of the plant matrix, favoring chemical and enzymatic modifications that can impair the product quality [24,46,47,48].

In general, frozen plant foods are characterized by lower textural properties than those of the initial raw material, and these changes could be mitigated by the application of process and storage conditions that limit the damages of the ice crystals as well as any other phenomena that could increase ice crystal size growth, like recrystallization [40,49].

Freezing and frozen storage can also affect the chemical properties of the plant products that could be modified with respect to those of the corresponding fresh foods by the damages induced by ice crystals on the tissue structure and cell integrity, as well as by the thawing conditions. Chemical and enzymatic reactions can cause loss or changes of some natural pigments (e.g., conversion of chlorophylls to pheophytins), formation of brown compounds due to enzymatic and oxidative reactions, degradation of carotenoids, or complexation of anthocyanins and other pigments [50,51,52]. Leaching phenomena upon thawing contribute to a further loss of solutes, micronutrients (e.g., salts, vitamins) and other small compounds.

As the antioxidant ability of a food depends on the presence and concentration of bioactive molecules able to hinder oxidative reactions, it is, thus, evident that all the chemical, physical and structural modifications induced by the frozen state of the plant tissue can, in turn, affect the antioxidant properties of the corresponding products. Moreover, freezing pre-treatments, like blanching, that inactivate endogenous enzymes, and the use of cryoprotectants able to limit physical and structural damages of the plant foods, are strategies applied to maintain the initial quality properties of fruits and vegetables during freezing. These actions, could thus affect the presence and concentration of bioactives, and their impact, either positive or negative, has to be disclosed in order to define the optimal freezing conditions to produce high-quality plant frozen foods.

Actually, the studies carried out on different plant foods that will be reviewed in the following sections highlighted that the antioxidant activity may remain constant or decrease or increase, with results that cannot be discussed simply by taking into account the nature of the plant product, the process conditions and the temperature of frozen storage.

## 3. Antioxidants and Antioxidant Activity of Fruits and Vegetables

### 3.1. Antioxidant Role of Fruits and Vegetables

Fruits and vegetables have a crucial role in the human diet and several reports have shown that an adequate intake is recommended for a healthy diet since it lowers the risk associated to chronic diseases such as several types of cancer, coronary heart disease (CHD), stroke and diabetes [5,53]. In this regard, vegetable products have always taken part in dietary guidelines with higher serving size than other food groups and recent guidelines recommended eating more vegetables than fruits, as they contain less sugar and are lower in calories [54].

The beneficial effects associated with fruit and vegetable consumption are generally attributed to their chemical composition since they provide health-promoting compounds such as nutrients, fiber and, in particular, phytochemicals [15,55,56], which are chemical compounds produced by plants to modulate their growth and defenses. Plant foods, given their fiber content, have long been considered as contributing to health promotion, but there are also indications of the potential benefits of the consumption of plant extracts because of their contents in nutrients and phytochemicals, with interesting bioactivity on human health [57]. Actually, many compounds are strong antioxidants and, even if present in low amounts, may counteract oxidation reactions. In human metabolism, the oxidative stress (imbalance between reactive oxygen species production and the neutralizing capacity of the antioxidant system), causing inflammation, represents a pre-pathological status and is a unifying hypothesis for a predisposition to chronic and degenerative diseases [56,58].

It has been noticed that the supplementation of the diet with galenic antioxidants cannot determine the same effect of increased food and vegetable, or plant extract intake [13]. This suggests that the beneficial properties for health that plant foods can provide are due to the co-presence of antioxidants with different mechanisms of action [59] and distribution between tissues and phases [15].

The antioxidants present in plant food could be classified as essential (ascorbic acid, tocopherols and tocotrienols and pro-vitamin A carotenoids) and non-essential (non-pro-vitamin A carotenoids, phenolic compounds, glucosinolates, organosulfur compounds, saponins, terpenes, lipoic acid, selected aminoacids or peptides and glucosamine) [53,60]. Phenolic compounds can be further grouped into phenolic acids, cumarins, flavonoids, tannins, calcones, stilbenes, lignans and cumestans. Flavonoids, in turn, can be divided into flavones, isoflavones, flavonols, flavanols, flavanones and anthocyanins. Finally, tannins could be grouped into hydrolysable tannins, condensed tannins, caffeates and ellagic acid [61]. Chlorophylls, that are constitutive compounds of green fruits and vegetables, also show antioxidant activity [62], but they are not reported among the antioxidants in the aforementioned classification.

Several classes of antioxidants are present in foods, and each antioxidant could exert a different protective effect towards oxidation. An integrated approach in measuring the food antioxidant potential could be more important than the concentration of every single compound [7]; therefore, in vitro and in vivo methods for the analysis of antioxidant activity (or capacity) of a food were developed [63].

The direct analysis of antioxidant activity by measuring the ability of food extracts to neutralize oxidants (radical oxygen or nitrogen species) or to counteract oxidation reactions is the most common analytical approach in food science. However, this analytical approach could underestimate the real in vivo effect of food consumption since it does not consider the contribution of some compounds (e.g., glucosinolates) to the increase of the antioxidant defenses of the human metabolism, that are mediated by enzymatically catalyzed pathways. Thus, some authors prefer to differentiate between the non-enzymatic antioxidant capacity (NEAC) and the real antioxidant capacity of a food.

The antioxidant activity of a food has been considered as an index of the potential health benefits associated to its consumption [64]. For this reason, the antioxidant capacity of plant foods has been evaluated by different in vitro assays [65] and fruits generally showed a higher antioxidant capacity than vegetables, with blue fruits rich in anthocyanins and proanthocyanidins and olives being those with higher antioxidant capacity. The antioxidant capacity ranking obtained by in vitro assays is different from that observed in vivo by measuring the antioxidant capacity of plasma [66,67] because some antioxidants (e.g., selected phenolic compounds or chlorophylls) are not available for intestinal absorption [68]. However, not bioavailable antioxidants could still act as sacrificial antioxidants at the gastrointestinal level [69], thus an integrated in vitro measurement of the antioxidant activity of a food could still be of interest in food research.

The measurement of the antioxidant activity or capacity of a food does not only have a functional significance but could also be used as an index to predict the oxidative stability of a food [70] or as an index of its changes or damage induced by processing [71,72,73].

### 3.2. Methods for Antioxidant Activity Determination and Main Criticisms

Many methods have been developed to measure antioxidant activity and they were extensively reviewed in the literature [74,75,76]. Hydrogen atom transfer (HAT)-based methods measure the ability of an antioxidant to quench free radicals by hydrogen donation, while single electron transfer (SET)-based methods detect the reducing ability of an antioxidant towards any compound, such as metals, carbonyls and radicals. In this paragraph, only the methods reported in the literature that tested the in vitro antioxidant activity of fruits and vegetables subjected to freezing and frozen storage will be briefly described and commented.

Undoubtedly, the most popular and widespread method is the Folin-Ciocalteu (FC) assay, whose mechanism is based on a redox reaction (SET) as polyphenols are oxidized in a basic environment by the FC reagent, which is a mixture of tungstate and molybdate with the formation of colored molybdenum ions [77]. Another SET test is the Ferric Reducing Antioxidant Power (FRAP), which, contrarily to the FC test, is carried out in an acidic environment needed for iron solubilization [78]. The antioxidant reduces the iron Fe(III) within 2,4,6-tri(2-pyridyl)-1,3,5-triazine (TPTZ) compound to get a colored product (Fe(II)-TPTZ). The Oxygen Radical Absorbance Capacity (ORAC) is a HAT-based method: it is a measure of the chain breaking activity of antioxidant compounds towards peroxyl radicals generated by thermal decomposition of azo-compounds in an aqueous buffer [79,80,81]. Two widely used antioxidant methods that are based on mixed SET and HAT mechanisms are the Trolox-Equivalent Antioxidant Capacity (TEAC) and the 2,2-diphenyl-1-picrylhydrazyl (DPPH) free radical assay, and both of them use a non-physiological radical. The TEAC is an assay, firstly reported by Miller et al. [82], that measures the reaction in an aqueous environment between antioxidant compounds and the colored and sTable 2,2′-azinobis (3-ethylbenzothiazoline-6-sulfonic acid) (ABTS+•) radical cation, which, upon reaction, undergoes discoloration. The method has been subjected to several modifications over time and the procedure more widely used is described by Re et al. [83]. In the DPPH assay, firstly reported by Brand-Williams and co-workers [84], a stable organic nitrogen radical, 2,2-diphenyl-1-picrylhydrazyl, is used. It is soluble in organic media and characterized by a deep purple color which, upon reaction, undergoes discoloration. Finally, the β-carotene bleaching test measures the decrease in the rate of β-carotene decay provided by antioxidants by monitoring the color loss in an aqueous oil-in-water emulsion made of linoleic acid and Tween 40. Radicals are usually generated by the spontaneous oxidation of linoleic acid [85].

Each method has its own advantages and disadvantages in terms of complexity, accessible equipment, chemical mechanism, conditions of analysis and quantification methods; however, what is absolutely relevant is the need for a continued effort to standardize each assay in order to get reliable and comparable results. Methods could also differ in the reaction medium (polarity, phase separation, pH, ionic strength, with reference to the above-mentioned methods), reaction time [86], as well as in the accessibility and the mechanism of action, and this enhances the difficulties related to the analyses and the interpretation of the results. In particular, ORAC and TEAC assays could be easily conducted in aqueous solutions at physiological pH (~7), whilst the FC and the FRAP methods are carried out in basic and acidic environments respectively, and the antioxidant activity of some compounds (e.g., polyphenols) could change dramatically depending on their protonation [87]. The β-carotene bleaching method is conducted in o/w emulsion. Oxidation reaction in emulsion could proceed in a different way than in a continuous phase [88] and the antioxidant activity of the aqueous phase is not strictly reflected in a lowering of oxidation rate in the lipidic dispersed phase [89].

The reaction time could definitely influence the results in an antioxidant activity test since some compounds could react almost instantaneously with radicals (e.g., ascorbic acid), whilst others are slow reacting antioxidants [86]; in many cases, sampling times lower than 10 min, depending on the type and concentration of the reactants, are more likely to yield the correct ranking of efficacy among different antioxidants [76]. The FRAP and ABTS tests require 4–6 min, whilst the ORAC, β-carotene bleaching method and DPPH could require longer times. Timewise, the Folin-Ciocalteu test requires 1 h of reaction.

As far as the medium polarity is concerned, DPPH is carried out in methanol; however, the bioactivity of antioxidant compounds (e.g., polyphenols) is influenced by the polarity of the medium [90,91]. DPPH can underestimate the antioxidant activity when aqueous extracts are used due to methanol–water phase separation in the media that could limit the contact between the water-soluble antioxidant and the methanol-soluble radicals [92]. It is interesting to point out that one of the most used methods in the literature for the evaluation of the antioxidant activity on plant foods and on the effect of freezing (see Tables 1–3), is the one with the most limitations.

The mechanism of action underlying the reaction between radicals and antioxidants is another factor responsible for discordance when the results obtained by different methods are compared. The reducing capacity and the radical scavenging activity of food extract are not always correlated among them [93]. Upon oxidation, the antioxidant activity of fruits and vegetables could show a decrease or even an increase depending on the chemical nature of the antioxidant and the mechanism of action underlying the reaction between the radical and the antioxidant itself. Generally speaking, ascorbic acid and carotenoids decrease their antioxidant activity and reducing power upon oxidation [78,94], whilst some phenolic compounds, such as the flavanols catechin and epicatechin, could increase their redox potential but increase their radical scavenging activity during oxidation [93], due to their polymerization up to tetramers, and then decrease it upon further polymerization [95].

The presence of metal ions in the extract could affect the antioxidant activity when some assays are used: chlorophyll-containing green vegetables have lower antioxidant capacity when the DPPH test is used; on the other hand, the results of the FRAP assay are influenced by Fe^3+^ ions’ presence in the plant extract. In addition, many large antioxidant compounds may slowly react with DPPH radical or may even be inert in this assay due to the steric inaccessibility of antioxidants to the radical [96,97]. As a large molecule, chlorophyll may not react with DPPH efficiently; therefore, the antioxidant capacity of chlorophyll-containing vegetables might be underestimated.

All the differences among the methods mentioned above could be a cause for discordant results when different methods are used to evaluate antioxidant activity; for this reason, it is always recommended to use more than one method.

## 4. Effect of Freezing and Frozen Storage on Antioxidant Activity (AOA) of Fruits and Vegetables

Freezing is a preservation technology widely used both by the food industry and in households for highly perishable and seasonal plant foods [98]. It is highly accepted by consumers since it does not require the use of preservatives and is retained to have a low impact on the nutritional quality of food products [21]. However, as previously discussed, plant foods can be damaged during freezing and frozen storage, and thus, the final quality of the products and its functional properties, especially after thawing, may be lower than the corresponding fresh product. Cell breakages can lead, in fact, to the decompartmentalization of antioxidants, such as anthocyanins and other phenolic compounds, and to their degradation due to the interaction with oxidative enzymes [99]. Moreover, soluble antioxidants can undergo possible exposure to oxygen and separation in the unfrozen phase in which oxidation reactions could proceed faster due to the increased concentration of the reactants [100,101]. Some authors have also shown that freezing operations may also exert positive effects on the quality and functional properties of plant foods, since after freezing, a release of bioactive compounds as bound phenolic acids and anthocyanins can occur, resulting in an increase of antioxidant activity [30,31].

In the literature, the studies focused on the evaluation of the effect of freezing and frozen storage on the antioxidant activity of fruits and vegetables in general report conflicting results. In order to highlight the effect of single and combined effects of freezing and frozen storage, the role of freezing pre-treatments and the influence of the confinement of antioxidants in cell structures on the antioxidant activity of plant foods, in this section, literature data were reviewed as a function of (i) type of plant food investigated, i.e., fruits or vegetable, (ii) the product structure, i.e., whole plant tissue or puree/juice, and (iii) the process applied, i.e., freezing or freezing and frozen storage in combination with eventual industrial thermal pre-treatments.

Research was carried out on major scientific databases such as Scopus, PubMed and Web of Science (WOS), and thanks to cross-references, 48 papers reporting AOA data of plant foods after freezing and/or frozen storage were found and reviewed. Among these, five were excluded based on the following criteria: (i) no clear information on the freezing method or the freezing temperature, (ii) missing information on the storage temperature and (iii) data duplicated in different studies. With respect to the resulting scientific papers, 25 were focused on fruits and 18 on vegetables.

### 4.1. Vegetables

#### 4.1.1. Effect of Freezing and Freezing Temperature

Studies performed do not always allow the evaluation of the impact of low temperatures on the AOA of vegetables, since the commonly used blanching pre-treatment can affect it much more than freezing [102,103]. Despite the positive effect on the retention of natural taste and flavors, the inactivation of native enzymes and the stabilization of a series of constituents of the raw material [98], blanching can impair the content of vitamin C and other thermo-labile antioxidant compounds as a consequence of leaching phenomena into the water and/or due to oxidation reactions [104]. Ninfali and Bacchiocca [105], evaluating total phenols and the antioxidant capacity by ORAC assay of beet green, spinach, broccoli, carrots and celery processed at the industrial level by blanching and freezing, highlighted, for instance, a decrease of the ORAC value in all the investigated vegetables except broccoli, and attributed these losses to the reduction of phenols (celery excluded) as an effect of the drastic blanching process imposed by the texture of the vegetables. A drastic AOA decrease due to blanching and freezing was also observed by Frati, Antonini and Ninfali [106], and by Hunter and Fletcher [102], on broccoli, spinach and peas analyzed by ORAC or FRAP assay. Conversely, Patras, Tiwari and Brunton [107], investigating the effect of blanching and blast freezing (−30 °C) on the retention of total antioxidant activity (DPPH) in blanched and unblanched broccoli, carrots and green beans, observed a decrease of the antioxidant activity as well as of vitamin C only on the unblanched samples.

When the effect of freezing has been evaluated by analyzing the antioxidant capacity data of blanched vegetables before and after the freezing process, no variation in the AOA was found in green and white cauliflower, kale leaves and broccoli florets [98,108,109,110]. These results highlight that if frozen foods are handled and processed properly, their functional properties can be retained during freezing. In particular, if enzymes that accelerate the oxidation of antioxidants such as vitamin C are inactivated by blanching, freezing causes negligible losses of these compounds [111].

Regarding the effect of the freezing temperature on vegetables, it is well known that it affects the freezing rate, which, in turn, influences the ice crystal size, and thus the food texture and the content of some bioactive compounds that may be lost due to leaching upon thawing [22,47]. The studies aiming to evaluate the effect of freezing temperature on the antioxidant activity of vegetables are scarce. Korus and Lisiewska [98] evaluated the AOA of kale leaves after freezing at two different temperatures (−20 and −30 °C) and showed no significant differences on the level of the antioxidants analyzed (polyphenols and vitamin C) nor in the antioxidant activity (TEAC) compared to the blanched sample. This same result was also found by Gębczyński [112] on green asparagus blanched and prepared for consumption.

#### 4.1.2. Effect of Frozen Storage and Storage Temperature

It is common practice to store frozen foods at temperatures equal to or lower than −18 °C, which is also the limit set by the law in some countries (such as the European Directive for Quick Frozen Foods). This storage temperature, while sufficient for prolonging the stability, is still above the glass transition temperature (Tg) of many plant foods. Consequently, during frozen storage, there can still be (bio)chemical activity and coarsening of ice crystals in the maximally concentrated solution fraction of the system [47].

Frozen storage of vegetables has shown controversial effects on AOA retention. In particular, Gebczyński and Lisiewska [113], and Gebczyński and Kmiecik [112] highlighted a negative effect of frozen storage on the AOA of both broccoli and cauliflower prepared for consumption mostly related to the parallel reduction of vitamin C and polyphenols, while β-carotene and carotenoids variations were very limited and/or statistically significant only after 12 months of storage. On the contrary, Hunter and Fletcher [102], on peas and spinach after the decrease of AOA due to blanching and freezing, did not observe further significant losses during storage at −20 °C. This effect was also observed by Korus and Lisiewska [98] on kale leaves stored for 12 months, despite the decrease of vitamin C and polyphenols, as well as by Kapusta-Duch et al. [109] in cauliflower stored at −22 °C for up to 3 months. Murcia, Jiménez and Martínez-Tomé [114], who evaluated the loss of AOA due to frozen storage (−20 °C for 8 months) on thirteen types of vegetables, evidenced different effects depending on both the vegetable and the method of analysis applied for the AOA determination.

The storage temperature can influence the quality of frozen plant foods by affecting ice coarsening and chemical kinetics. Temperature, in fact, affects the kinetic energy of molecules and their diffusion in the unfrozen phase, but also the viscosity of the unfrozen system, which in turn can influence the dynamics of high molecular weight biomolecules such as enzymes. Protein dynamics, more than water diffusion, can become the rate-limiting factor for enzymatic reactions [115].

Few studies and discording results were found in the literature on the effect of the storage temperature on the AOA of vegetables. Korus and Lisiewska [98] did not notice significant differences on the TEAC value of kale leaves stored at −20 and −30 °C despite the decrease of vitamin C and polyphenols, which occurred at different extents at the two storage temperatures (−19% vs. −11% for Vitamin C and −14% vs. −10% for polyphenols). Conversely, Gebczyński and Kmiecik [112] and Gebczyński and Lisiewska [113], investigating the antioxidant activity by DPPH assay of cauliflower and broccoli prepared for consumption during frozen storage at −20 and −30 °C, observed a negative effect of the storage temperature. Storage at −30 °C, in fact, allowed the preservation of the antioxidant compounds (vitamin C, carotenoids, β-carotene and polyphenols) and led to the highest antioxidant activity values (+9% in cauliflower).

#### 4.1.3. Combined Effect of Freezing and Frozen Storage

Literature studies reporting AOA data for blanched vegetables after freezing and frozen storage show that the effect due to processing is generally species- and variety-dependent and results are often affected by the method of analysis used for the AOA evaluation, as described and discussed in Section 3.2. Moreover, the different loss of integrity of tissues, cells, membranes and organelles induced by different freezing pre-treatment and their combination with freezing and frozen storage can differently affect the extractability of antioxidants during frozen storage [116].

For instance, Puupponen-Pimiä et al. [103] evaluated the effect of industrial blanching/freezing and long-term freezer storage on peas, carrots, brassicas, spinach, potato and swede, and observed that the effects on the antioxidant activity were species-dependent and mostly related to the phenolic fractions. In particular, no effects were observed on carrots, brassicas, spinach and potato, whereas a slight decrease of the AOA was observed on peas and swede for long storage times. Also, Murcia et al. [114] evaluated the loss of AOA of thirteen types of vegetables subjected to industrial freezing and highlighted different effects depending on both the vegetable and the method of analysis applied for the AOA determination. Volden, Bengtsson and Wicklund [117] investigated the effect of freezing and one year of freezer storage on the AOA of different varieties of cauliflower (*Brassica oleracea* L. ssp. botrytis) pre-treated by blanching and found changes only on some varieties and at the end of the storage period, with an average reduction of FRAP and ORAC values of 15% and 37%, respectively. These results were mostly related to the decrease of the vitamin C (from 4% to 28%, depending on variety) and, to a limited extent, of the total phenol content. It is important to highlight that in this study, the data are reported on wet basis and, thus, results may be influenced by the concentration effect due to the loss of internal water at thawing.

Bulut et al. [48] evaluated the effect of frozen storage (−27 °C) in a home-type freezer on the quality parameters of blanched green beans for 14 weeks and highlighted no significant effect on both the AOA and TPC of the vegetables, whilst the vitamin C, whose extractability was enhanced by the freezing process, steadily decreased after 7 weeks of frozen storage. Wolosiak, Druzynska, Piecyk, Worobiej, Majewska and Lewicki [118], studying the influence of industrial freezing and one year of frozen storage on AOA of green peas and string beans, observed different effects depending on the method of analysis used for the determination of the AOA (DPPH, ABTS or Fe^2+^ chelating properties).

In general, the studies carried out on unblanched vegetables show the highest variations of the antioxidant activity during frozen storage due to endogenous enzymes (e.g., polyphenoloxidase, peroxidase, lipoxygenase, ascorbic acid oxidase, chlorophyllase), whose activity is only slowed down by the low storage temperatures. Moreover, in most cases, the extent of these variations seems to depend on the vegetable composition. Martínez, Pérez, Carballo and Franco [119], studying the effect of freezing and frozen storage on both blanched and unblanched turnip, observed a decrease of the antioxidant activity (DPPH assay) occurring to the highest extent in the unblanched samples and related to the degradation of phenolic compounds or vitamin C. The decrease of AOA was also observed by Loizzo, Pugliese, Bonesi, Menichini and Tundis [120], on twenty cultivars of unblanched chili peppers after 4 months of home freezing (−20 °C), while Danesi and Bordoni [16], after home freezing and storage (6 months at −18 °C), observed on fresh carrots and yellow peppers a decrease of the AOA (ABTS assay), no variations on fresh tomatoes and an increase of AOA in fresh peas, zucchini and green beans. The authors ascribed their results to the different content of vitamins, carotenoids and phenolic compounds, which act synergistically but are differently sensitive to processing.

By comparing all the results reported in different literature studies (Table 1) on the effect of freezing, and of freezing and frozen storage on antioxidant activity of vegetables, with vegetable product being equal, in general, no effects were highlighted for green beans, kale leaves and cauliflower after both freezing and frozen storage, while on spinach, a negative effect was observed just after freezing. For carrots, broccoli, Brussels sprouts and peas, discordant results were among the reviewed studies, which can be ascribed to the different methods of analysis of the AOA.

### 4.2. Fruits

The preservation of fruits by freezing has become one of the most important preservation methods to extend the usage of frozen fruits during the off-season and to access remote markets that cannot be reached with fresh fruit. Frozen fruits are especially used by the processing industries for the production of juice, jams, canned products, as well as for the production of dessert, dairy and bakery products, etc., while the direct consumption by consumers is less frequent. Drip loss, enzymatic browning and softening occurring during freezing and thawing processes of fruits (e.g., apples, melon, peaches, apricots, etc.) as a consequence of ice crystal formation and cell disruption are the main phenomena responsible for undesirable physico-chemical changes and loss of bioactive compounds in frozen fruits. Unlike vegetables, most frozen fruits before freezing are not pre-treated by conventional thermal treatments, i.e., blanching, thus the effects on antioxidants, softening and color changes evidenced after freezing and frozen storage are promoted by the enzymatic reactions, which are only slowed down by the low storage temperatures.

Studies reported in the literature on the effects of freezing and frozen storage on the AOA of fruits refers both to whole fruits or pieces and puree or juices. In order to highlight the role of the confinement of antioxidants in cell structures on the AOA of frozen fruits, in the following sections, the effects on structured and de-structured products have been discussed separately.

#### 4.2.1. Effect of Freezing and Freezing Temperature

In general, the studies carried out on the effect of the freezing process on the AOA of whole fruits either report no variation or slight variations. Some authors [21,30], after liquid nitrogen freezing, observed no change in the concentration of phenols, vitamin C and AOA of raspberries and strawberry. The same result was also observed by Poiana, Moigradean, Raba, Alda and Popa [122] after the Individual Quick Freezing (IQF) process, and by other authors on blueberry, red raspberry, blackberry, serviceberry, makiang and maluod [123,124,125] frozen at higher temperatures.

Jiménez, Martínez-Tomé, Egea, Romojaro and Murcia [126] reported that freezing did not cause significant changes in the capacity to scavenge OH^•^ radical, but it slightly decreased the O^2•−^ scavenging of blanched apricots. González, De Ancos and Cano [127], after freezing at −80 °C, observed a slight decrease of the AOA in blackberry and in some raspberry cultivars. Marques et al. [128] observed a decrease of AOA on strawberries frozen at −20 °C.

In general, the freezing process negatively affects the AOA of fruit puree or juices. Gonçalves et al. [129], after freezing of strawberry pulp, determined a decrease of the AOA by both DPPH and ABTS on both pasteurized and unpasteurized puree, irrespective of the freezing method applied (−25 °C by forced air or −18 °C by static air). Patthamakanokporn et al. [125], after freezing of guava puree at −20 °C, observed a decrease of the AOA by both the ORAC and FRAP assay as well as of the polyphenols content, and this same effect was also observed by Loncaric et al. [130], after freezing (12 h; −18 °C) of apple puree (cv. Idared and Fuji) by the ABTS and DPPH assays. However, Loncaric et al. [131], on frozen apple puree of different apples, cv. Granny Smith (GS) and Gold Rush, despite the polyphenol decrease, did not observe AOA loss due to freezing (24 h; −18 °C).

The observed effects on whole fruits and juices or puree depend on the different structure of the food. Fruit disintegration by homogenization or juice extraction before freezing can promote, in puree and juices, the reaction of the endogenous oxidase enzymes with consequent reduction of the antioxidants level.

#### 4.2.2. Effect of Storage and Storage Temperature

Frozen storage of fruits has shown controversial effects on AOA retention. In particular, conflicting results were found, even with the same fruit, depending on the test used to determine the AOA.

By investigating the effect of frozen storage (−18 °C up to ten months) on the AOA of blueberry, red raspberry and blackberry frozen by IQF, Poiana et al. [122] observed a loss of the antioxidant activity in all the investigated fruits with blueberries and raspberries showing, respectively, the lowest (approximately 23%) and the highest (approximately 37%) values, and with a correlation coefficient between FRAP and the total phenolics higher than the one between FRAP and total anthocyanins or FRAP and vitamin C for all investigated fruits. Conversely, González et al. [127], during frozen storage for one year at −24 °C, observed no AOA variation both in different raspberry cultivars and on wild blackberry. Michalczyk and MacUra [124], on serviceberry stored at −20 °C for 10 months, observed no variation, or an increase of the AOA, depending on the method applied for the AOA evaluation (respectively DPPH assay or Fe^3+^ reducing power). Patthamakanokporn et al. [125] observed different rates and degrees of loss in the ORAC and FRAP antioxidant activity during the storage of homogenized guava, intact maluod (small, soft flesh fruit) and makiang (small, firm flesh fruit). The ORAC antioxidant activity in the homogenized guava and the intact maluod decreased significantly during storage at −20 °C for 2 weeks and continued to decrease during 3 months of storage, whereas that of makiang was stable. In contrast, FRAP antioxidant activity did not decrease in homogenized guava, but those of the whole fruits of makiang and maluod decreased.

The effect of the storage temperature on the AOA retention of frozen fruits is unclear and seems to be mainly related to intrinsic factors related to the vegetable species. Ścibisz and Mitek [123] did not show any difference in the evolution of the TEAC values, anthocyanin and total polyphenol content in blueberry fruits stored at −18 and −35 °C for 6 months, and this same effect was also found by Khattab et al. [99] on Haskap berries at both −18 and −32 °C. Conversely, a negative effect of storage temperature on AOA was found by Chaovanalikit and Wrolstad [51] on sour and sweet cherries stored at −23 and −70 °C for 6 months after freezing with liquid nitrogen. In particular, a decrease in antioxidant activities (28% for ORAC and 58% for FRAP) during 6 months of storage at −23 °C was found along with the loss of anthocyanin and polyphenols, whereas there was an apparent increase in antioxidant activities for cherries stored at −70 °C (81% for ORAC and 46% for FRAP). Since the reduction in antioxidant activities for cherries stored at −23 °C was not nearly as high as the losses in total anthocyanins and total phenolics, the authors hypothesized that anthocyanin and polyphenolic degradation products retain antioxidant activities. The effects observed at −23 °C on anthocyanin and polyphenols degradation in frozen cherries were likely related to the presence in both flesh and skin of native enzymes, particularly polyphenoloxidase, which accelerate anthocyanin degradation in the presence of polyphenols, particularly chlorogenic acid, one of the major phenolic compounds in this fruit. Kader and others [132] showed, in fact, that in blueberries, polyphenoloxidase oxidized chlorogenic acid to a quinine, which couple with anthocyanins in a degradation reaction. Chlorogenic acid is then partially regenerated and can continue to serve as a substrate for polyphenoloxidase.

It needs to be remarked that at −70 °C, being the unfrozen phase in cherry below its glass transition temperature and thus, in glassy state, all diffusion-limited reactions, including enzymatic activity, could not occur because they were hindered by the reduced molecular mobility of the system.

However, Sacchetti et al. [31], during storage at −18 and −80 °C of strawberries frozen in liquid nitrogen, observed a decrease of the antioxidant activity of about 30% at −80 °C and an increase up to 50% at −18 °C (dry basis). Accordingly, antioxidants (ascorbic acid and polyphenols) reflected the same trend, with an 8% reduction after 8 months of storage at −80 °C and an increase of antioxidants (20–25%) during storage at −18 °C. The authors attributed the results of antioxidant activity and of antioxidant compounds observed at −18 °C to the higher accessibility of antioxidants for extraction due to freezing damage. The microcrystals of ice formed during rapid freezing in liquid nitrogen once conditioned at −18 °C, undergo melting and recrystallization phenomena with an increase in their size [25], with consequent rupture of the membranes and cell walls, which allows greater extractability of intracellular antioxidants.

As concerns the effect of storage temperature on puree and juices, Polinati, Faller and Fialho [133] did not evidence variation in the antioxidant capacity and in the polyphenol content in apple puree and orange juice frozen both at −18 and −70 °C for up to 10 days. Conversely, Gonçalves et al. [129], during storage at −18 °C of strawberry pulp, highlighted no effect or decrease of AOA depending on the method and temperature of freezing applied (−25 °C by forced air or −18 °C by static air) and on the use of pasteurization as a pre-treatment to freezing. In general, a higher AOA retention in strawberry pulp frozen by fast air was noted when compared to that frozen by static air by both the DPPH and ABTS assay.

#### 4.2.3. Combined Effect of Freezing and Frozen Storage

Due to the high variability of the results reported in the literature, it is not possible to identify the effect due to the combined effect of freezing and frozen storage on the AOA of whole fruits.

Bulut et al. [48] evaluated for 14 weeks the effect of frozen storage (−27 °C) in a home-type freezer on the quality parameters of strawberry, and besides the decrease of vitamin C after 7 weeks of frozen storage, no significant effect on both the AOA and TPC of the fruits was noticed. The same results were also observed on blueberries frozen and stored for three months at −20 °C by Lohachoompol, Srzednicki and Craske [134]. The latter result was in disagreement with that observed on the same fruit by Reque et al. [52], who analyzed the antioxidant activity and the anthocyanin stability of blueberry frozen and stored at −18 °C for 6 months, highlighting a positive effect of frozen storage by both ABTS and DPPH assays, despite the significant losses of anthocyanins caused by oxidation and/or condensation reactions with other phenolic compounds. This difference allowed the authors to infer that the other phenolic compounds, or an association of these with the anthocyanin degradation products, were responsible for maintaining the antioxidant activity and even increasing it during storage. On the other side, Kopjar, Tiban, Pilizota and Babic [135], investigating the effect of frozen storage at −18 °C on two different blackberry cultivars, observed a loss of AOA (8% on average), related to the loss of both polyphenols and anthocyanins.

Other studies highlighted a negative effect of freezing and frozen storage on the antioxidant properties of fruits as a result of the concurrent loss of such antioxidants. In particular, it was also observed on Haskap berries (*Lonicera caerulea* L.) [99] and on sour and sweet cherries cultivars [51]. Also, Blanda, Cerretani, Bendini, Cardinali and Lercker [136], on nectarine frozen at −80 °C for 15 min and stored at −18 °C for one month, observed a decrease of the TEAC values. These negative attributes were probably linked to the activity of polyphenoloxidase (PPO) and depletion of phenols due to cell rupture during freeze–thaw procedures. Similar results were also observed by Santarelli et al. [73] on both conventional and organic apples (cv. Golden Delicious) frozen and stored at −40 °C for 10 months, and by Freire, De Abreu, Rocha, Corrêa and Marques [137], on acerola and guava fruits evaluated by both ABTS and DPPH assays after 3 months at −18 °C. The same authors in the same study observed discordant results between the method of analysis applied for the AOA determination for both cashew and strawberry.

As regards the combined effect of freezing and frozen storage on juice or puree, generally, a decrease of the antioxidant activity is evidenced. Mirsaeedghazi, Emam-Djomeh and Ahmadkhaniha [138], evaluating the antioxidant activity in pomegranate juice stored at −25 °C for up to 20 days, observed a decrease of the DPPH radical scavenging properties related to the reduction of the major antioxidant components, such as anthocyanins and phenolic compounds, due to oxidation reactions. Loncaric et al. [130], evaluating the effect of frozen storage (6 months at −18 °C) on the AOA of apples’ (cv. Idared and Fuji) puree, obtained different results depending on the method of analysis applied. In particular, the results obtained by the DPPH method indicated a decrease of AOA during storage of the samples of both apple varieties, whilst the ABTS assay highlighted no effect for the cv. Idared and an AOA increase for the cv. Fuji.

Comparing the results reported in different literature studies (Table 2) on the effect of freezing, and of freezing and frozen storage on antioxidant activity of fruits, with fruit product being equal, an AOA decrease was found for hasak berries, blackberries and apple puree after both freezing and frozen storage, no variation was observed in orange juices, while in general, discordant results were found among the reviewed studies for whole strawberries, raspberries and blueberries. These discrepancies were also found irrespective of the method of analysis used to determine the AOA and could depend on process-related factors such as the thawing methods and wounding [139,140] and/or on factors related to the raw material. In fact, the cultivar, the cultivation method, the ripening degree, the harvest season and pre-harvest or postharvest chilling stresses can significantly affect the composition of plant tissues and, thus, their mechanical strength [141] and response to physical stresses induced by processing, with effects on the release and oxidation of polyphenols after freezing and thawing [22,73,142]. In particular, these variables influence the composition of plant foods by affecting (i) the content of proteins and of sugars, which act as natural cryoprotectants [143], (ii) the phenolic content and composition due to the shift of the pathways involved in their biosynthesis [139,144,145,146] and the concentration of oxidase enzymes, such as polyphenoloxidase, whose activity is affected not only by the total polyphenol content but also by the content of single polyphenols and/or by their oxidation products [147].

## 5. Cryoprotectants and Their Effect on Antioxidant Activity of Frozen Plant Products

Freezing allows extending the shelf-life of vegetables and fruit products, preserving most of their original attributes and extending their availability throughout the year, however, because of the ice formation, deleterious effects occur in the frozen food matrix [40]. Besides the improvement and optimization of process parameters and technology used during freezing, another strategy adopted to limit deteriorative changes in food products is the use of cryoprotectants. The term ‘cryoprotectant’ accounts for a wide class of compounds that ranges from small saccharides to inorganic salts, proteins, hydrocolloids and other molecules able to prevent damages caused by the freezing process. Cryoprotectants can be naturally present in foods or added during processing and formulation [33]. Their application depends on their intrinsic physicochemical properties, type of freezing technology used and the food matrix composition where they are added [36].

Cryoprotectants are classified in two different classes according to the stabilization exerted, which could be based on colligative or non-colligative type mechanisms. When the mechanism of cryoprotection is based on the variation of product liquid fraction properties, such as freezing point or osmotic pressure by the solutes, it is considered a colligative-based mechanism. In this case, the stabilization is not dependent on the type of molecule but only on its concentration. Conversely, the stabilization mechanism based on non-colligative effects is strictly related to the features and structural peculiarities of the cryoprotectants [35,150].

### 5.1. Colligative Type

Sugars, which belong to the colligative-type cryoprotectants, are one of the most used to prevent cold damages during freezing and frozen storage, especially in fruits, while salts are more commonly applied in vegetables, with a significant improvement of the final quality of the products [36]. While these solutes can be easily added in formulated preparations (e.g., purees, juices), the addition in entire pieces or parts of cellular foods (e.g., slices and cubes) remains more difficult because it requires the penetration of the cryoprotectant into the product, mediated by a mass transfer mechanism. In food processing, this is usually performed by technologies like osmotic treatments and vacuum impregnation [151] that are applied as pre-treatments before freezing. In particular, in the dehydrofreezing, osmotic treatments are carried out before freezing, and the food, whole or in pieces, is dipped in a hypertonic solution. The different osmotic pressures between the two systems allows the solutes’ penetration throughout the product [33,152].

The presence of cryoprotectant solutes in fruit and vegetables reduces the damage extent of vegetal tissues by reducing the drip loss and allowing higher retention of bioactives, original color and aroma, and thereby a higher preservation of the product’s original quality is guaranteed [36,40]. Moreover, it has been observed that sugars in frozen vegetable matrices hamper the oxygen from diffusing inside the product, limiting oxidation that could impair product appearance such as browning phenomena [153].

The mechanism of cryoprotection of these types of solutes is based on a reduction of the free water (i.e., aw) in the cells, with consequent depression of the freezing point and concurrent increase of the glass transition temperature of the product frozen maximally concentrated solution (Tg’) value. As long as the product is kept below its Tg’, all the deteriorative processes based on diffusion are significantly slowed and the product is more stable [154,155]. Moreover, sugar molecules showed the ability to affect the ice formation process by modulating the ice crystal growth inside and outside the cells with an effect that depends on their molecular weight [45].

Inorganic salts (e.g., NaCl) are used to a limited extent due to their main impact on the taste of plant products and potential damages on chloroplast thilakoids membranes [35]. However, the pre-treatment of vegetables intended for freezing with calcium salts is particularly relevant as it results effective in the preservation of the product texture. Calcium ions’ interaction with pectic acid moieties leads to the formation of calcium pectate, which confers firmness to the vegetal tissue cells [156,157,158].

Besides small saccharides and inorganic salts, polyols have also been employed in freezing pre-treatments with similar benefits on the product final quality [45,159], and James et al. [36] provided an exhaustive list of the application of these solutes in fruits and vegetables via osmotic dehydrofreezing.

### 5.2. Non-Colligative Type

Hydrocolloids, polymers such as polysaccharides and proteins are a second class of non-colligative cryoprotectants able to stabilize frozen products, hindering recrystallization, phase separation, moisture migration or volume shrinkage during storage [160]. Those molecules are characterized by a rather high molecular weight, hydrophilicity and are able to affect rheological and textural properties of food systems [161]. The use of hydrocolloids in freezing pre-treatments of fruits and vegetables leads to a reduction of turgor loss in plant tissue and higher retention of bioactives and nutritional compounds [162,163,164,165]. Hydrocolloids have also been successfully tested in frozen formulated vegetable products (e.g., mashed potatoes) with significant improvement of the product texture, creaminess, flavor and color retention and inhibition of starch retrogradation [166,167,168,169,170]. In some cases, their application has been coupled with sugars or calcium salts for an improved cryoprotectant effect, with a consequent increase of product freezing velocity and a reduction of textural and drip loss [165,171]. The cryoprotectant ability of hydrocolloids, not fully understood yet, has been proposed to be related to their hydro-dynamical properties, i.e., their high water-holding capacity, the reduction of water mobility of the systems and the ability, in vegetable matrices, to directly interact with plant tissue cells, preventing damages upon biological structures [161,163,172]. However, the cryoprotectant activity exerted by hydrocolloids is strongly dependent on the type of food matrix they interact with [166].

A different class of non-colligative cryoprotectants is represented by ice structuring proteins, also known as “antifreeze proteins”, able to prevent cold damages on biological cells by affecting the ice crystallization habit process, inhibiting the recrystallization and thermal hysteresis [173]. These proteins, which have been isolated from fish, plants, insects, fungi and bacteria, do not have any structural peculiarity in common apart from the presence of a certain number of flat, slightly hydrophobic regions, which are identified as ‘ice binding sites’. They are able to depress water freezing point from 1 to 5 °C, depending by the type of organism they have been synthetized from without changing ice melting temperature (0 °C). In particular, when these proteins are in a supercooled water state, they locate themselves at the molten/solid interface (i.e., water/ice) with a mechanism which has not been clearly explained yet but is apparently related to the reduction of the ice–water interface area at which correspond a lower system overall energy, thus a thermodynamically favored state. Nowadays, six different types of ice structuring proteins have been identified: “hyperactive antifreeze proteins”, found in insects, showed the highest freezing point depression, becoming the most interesting and the most investigated among the different types of proteins lately. The presence of ice structuring proteins at the water–ice interface causes an inhibition of ice crystals’ growth, their agglomeration into larger clusters and hindering ice recrystallization that could be triggered by temperature fluctuation during frozen storage [174,175]. From the limited applications in fruit and vegetable matrices subjected to freezing reported in the literature, these proteins resulted in a reduction of drip loss and a better preservation of original texture, color and antioxidant compounds [176,177,178,179,180,181]. On the contrary, ice structuring proteins have been widely tested and successfully applied in ice cream and frozen dessert productions with promising results [182], even if their industrial application is not very common due to some drawbacks. In particular, some ice structuring proteins that have been tested and applied in food formulations, are genetically modified, leading to a reduced consumers’ acceptance; moreover, direct and indirect costs of purification and isolation or synthesis still remain high for the industry, limiting their applicability in food formulations [183,184].

### 5.3. Effect of Cryoprotectants on Antioxidant Activity

Despite the high number of publications that investigated the effectiveness of cryoprotectants on bioactive compounds, very few studies evaluated the impact on the AOA of the products and could thus be included in this review.

In Table 3, a comprehensive list of publications, which investigated the variation of antioxidant capacity in fruits or vegetable matrices pre-treated with the addition of cryoprotectants subject to freezing, is reported. Most of the studies that investigated the relationship between use of cryoprotectants and the AOA focused on the use of sugars applied on whole fruit or fruit-formulated preparations. Sugars resulted as having a positive impact on the AOA of sweet [185] and sour [186] cherries, blackberries [135], apple [130,131] and arazá [187].

Sweet cherries subjected to osmoblanching in a mixture of glucose:fructose (2:1 ratio) and stored at −20 °C showed significantly higher radical scavenging capacity (DPPH radical) compared to control samples, which were not treated with cryoprotectant compounds [185].

The effect of glucose and fructose, along with sucrose, on AOA was also studied by Kopjar et al. [135] in blackberries, blended with sugars, and stored at −18 °C for 11 months. Over the storage time, the reference sample, with no sugars added, showed a higher decrease of DPPH radical scavenging activity (≂8%) than samples treated with glucose and fructose, or with a mixture of the two sugars (1:1 ratio). Glucose resulted in being the best cryoprotectant in terms of AOA for blackberries, while sucrose resulted in being unfavorable with a residual DPPH scavenging ability lower than reference samples. A similar result was observed in sour cherry puree subjected to freezing and freeze-drying, added with sucrose at different concentrations, that impaired the sample AOA as well. The only saccharide that exerted a positive effect on the AOA of the fruit formulation was maltose at the highest concentration (20% *w*/*w*), while lower values of AOA, assessed by ABTS and FRAP tests, were also observed for trehalose [186]. Conversely, in arazá puree, thermally processed and then stored at −20 °C, the addition of sucrose at 20% (*w*/*w*) better preserved sample AOA compared to control in all the different assays performed [187].

In apple purees, prepared with different variety of apples, frozen and freeze-dried, the effect of the addition of glucose, fructose, trehalose and sucrose at concentrations of 1% and 5% (*w*/*w*) was studied and the two disaccharides were reported to be the more effective in the preservation of AOA during both the freezing and freeze-drying stages [130,131].

In the studies reported above, some disagreement in the results of the cryoprotectant effect was also observed depending on the type of AOA assay used. It has been shown how the presence of both sugars and phenolic compounds in the system can affect the results of AOA assay based on the formation of radicals [186,187,188,189].

The effect of a different type of cryoprotectant on AOA was scarcely investigated and, to our knowledge, the only paper available is that written by Kong et al. [178]. The DPPH radical scavenging ability was determined in cherries stored at −20 °C for 31 days pre-treated with three different ice structuring proteins. The addition of the antifreeze proteins effectively preserved cherry samples AOA compared to the reference samples. The residual AOA was correlated with content variation of anthocyanins and phenols as well [178].

To avoid misinterpretation of the results, many studies coupled the determination of AOA with the evaluation of the presence and quantification of the antioxidant compounds. A correlation between AOA values and concentrations of phenolic compounds, flavonoid compounds and anthocyanins has been found in fruits and vegetables, but not with vitamin C [127,135,186,190,191,192,193] (Table 3).

As already reported, phenolic compounds, particularly flavonoids, are considered key compounds responsible for the AOA of fruits. Changes in their content and composition in vegetal matrices are usually correlated to the product AOA trend, especially in the frozen storage phase more than the solely freezing step. However, the effect of cryoprotectants on the different classes of antioxidant compounds present in fruits and vegetables remains difficult to be univocally described. The wide range of compounds either used as cryoprotectants or as antioxidant molecules present in fruits and vegetables could represent one of the main, but not the only, hurdles to a clear definition of the cryoprotectant mechanism. Variations of antioxidant compounds’ relative composition in vegetal products of the same cultivar, caused by different degrees of ripening, lead to discordant results on the use of specific cryoprotectants towards anthocyanins. In particular, the use of sugars as cryoprotectants towards anthocyanins molecules in strawberries led to contradictory results according to factors such as the processing, matrix type (portion of whole fruit vs. fruit juice or puree), sugar type and concentration [194]. On the other side, Loncaric et al. [186] reported a negative effect caused by the addition of sucrose in sour cherry puree, probably due to the low a_w_ value of the sample which inhibited the interaction between the sugar molecule and the antioxidant compounds.

Hence, the high number of variables which can play an active role on the modulation of cryoprotectants’ activity and their synergic interaction makes it difficult to predict the effectiveness of specific compounds towards single classes of bioactives, or even molecules. To date, the only effect generally observed when cryoprotectants are used is the positive correlation between the reduction of the drip loss and the retention of water-soluble antioxidant bioactives.

## 6. Final Remarks and Perspectives

The review of the literature on the effect of freezing on the AOA of plant foods highlighted that it depends on several factors, such as type of plant product, nature and concentration of antioxidants present in the food, freezing process parameters and technologies, frozen storage temperature and thawing conditions. Moreover, by comparing the trends of AOA of the same plant product having different integrity, i.e., whole tissue, puree or juice, different effects of the same factors on AOA during both freezing and frozen storage could be observed. In particular, in whole fruits, the release of bioactives due to cell structural damages associated to the water crystallization can occur and this can induce either an increase (if they remain in the product) or a decrease (if leached during thawing), or even remain unchanged. On the other hand, fruit disintegration by homogenization or juice extraction before freezing can favor chemical and enzymatic reactions with consequent reduction of the antioxidants level in the corresponding puree and juices.

Factors of different nature could be considered as causes for these discordant effects highlighted for some fruits and vegetables. As remarked in Section 3.2, there are several different methods of analysis available for the evaluation of AOA, whose results depend on the presence of different classes of bioactives or their mechanism of action. Moreover, literature data have often been reported on wet bays and, thus, some results could have been influenced by the leaching at thawing and loss of water. Data expression on dry weight is, in fact, a fundamental methodological approach that allows the comparison of literature data related to the bioactives content of frozen plant foods and to their antioxidant activity.

Additional factors that could highly influence the AOA of frozen plant products, and that have been considered only to a limited extent in the studies reported in the literature, are the intrinsic characteristics of fresh product related to the agronomic practices and climate conditions during cultivation, as well as the plant variety or cultivar. These factors in general affect the initial content of antioxidants along with the plant tissue, cell walls’ composition and physical characteristics, and, in turn, the resistance of the product to the freezing stresses due to volume expansion and ice crystallization during freezing.

One factor that is scarcely considered, especially in fruits, and that can affect the response of plant foods to thermal stresses and the retention of the antioxidant activity is the ripening degree. The ripening degree, in fact, besides its effect on the structural properties, influences the content of bioactives, of natural cryoprotectants such as sugars and also the enzymatic activity. Scarcely considered are also all the post-harvest conditions that in general are not reported, and that, in combination with other factors, can lead overall to a large diversity of the AOA in frozen plant products.

All these underestimated factors offer new opportunities of future investigation to expand knowledge in this field, but also to identify the optimal conditions and product characteristics needed to improve the quality and healthy properties of frozen products.

Finally, new freezing technologies (e.g., impingement, hydrofluidization, ultrasound-assisted, pressure-shift), despite their relevance and interest in the sector, were not discussed in this review as they have been scarcely investigated regarding their effects on the bioactive pattern and the AOA of the plant foods. Studies on their ability to preserve quality properties and AOA of the frozen products could be of interest for their future development at the industrial scale.

## Figures and Tables

**Table 1:** Single and combined effect of freezing and frozen storage on the antioxidant activity of fruits.

												Effects				
Vegetable	Freezing System	Freezing T (°C)	Storage T (°C)	Storage Time	Pre-Treatment	Post-Process	AOA Test					Freezing	Storage	Freezing and Frozen Storage	Data Expression	References
beet green	flo-freezer	nr	ns		blanching					ORAC		decrease			fw	[105]
broccoli	nr	−40	−20	6, 12, 18 months	blanching			DPPH					no effect		dw	[103]
	blast chamber	−40	−20	4, 8, 12 months	blanching	cooking		DPPH						decrease	fw	[113]
	IQF	−26	ns		blanching				FRAP			no effect			dw	[108]
	industrial freezing	nr			blanching					ORAC		decrease			dw	[106]
	blast freezer	−30			blanching			DPPH				no effect			dw	[107]
	blast freezer	−30			untreated			DPPH				decrease			dw	[107]
	nr	−22			blanching		ABTS					no effect			fw	[110]
	flo-freezer	nr	ns		blanching					ORAC		no effect			fw	[105]
brussels sprouts		−35	−18	2, 4, 6, 8 months	blanching	boiling		DPPH						DRP	fw	[116]
	nr	−22			blanching		ABTS					decrease			fw	[110]
cabbage	nr	−40	−20	6, 12, 18 months	blanching			DPPH					no effect		dw	[103]
capsicum varieties	home	−20	−20	4 months	untreated		ABTS	DPPH	FRAP		β-CB			decrease	fw	[120]
carrots	flo-freezer	nr	ns		blanching					ORAC		decrease			fw	[105]
	nr	−40	−20	6, 12, 18 months	blanching			DPPH					no effect		dw	[103]
	blast freezer	−30			blanching			DPPH				no effect			dw	[107]
	blast freezer	−30			untreated			DPPH				no effect			dw	[107]
	home freezer	−18	−18	6 months	untreated		ABTS							decrease	fw	[16]
cauliflower	chamber freezer	−22	−22	24 h + 1, 2, 3 months	blanching		ABTS					no effect	no effect		dw	[109]
	nr	−40	−20	6, 12, 20 months	blanching			DPPH					no effect		dw	[103]
cauliflower	freezer	−24	−24	3, 6, 12 months	blanching				FRAP	ORAC				DRM	fw	[117]
	nr	−22			blanching		ABTS					no effect			fw	[110]
	blast freezer	−40	−20	4, 8, 12 months	cooking			DPPH						decrease	fw	[121]
celery	flo-freezer	nr	ns		blanching					ORAC		decrease			fw	[105]
green asparagus	chamber freezer	−20	−20	4, 8, 12 months	blanching	boiling		DPPH				decrease	decrease		dw	[112]
	chamber freezer	−30	−30	4, 8, 12 months	cooking	heating		DPPH				decrease	decrease		dw	[112]
green beans	home freezer	−23	−27		blanching			DPPH						no effect	fw	[48]
	home freezer	−23	−27		blanching			DPPH						no effect	fw	[48]
	industrial freezing	nr			blanching					ORAC		no effect			dw	[106]
	blast freezer	−30			blanching			DPPH				no effect			dw	[107]
	blast freezer	−30			untreated			DPPH				no effect			dw	[107]
	home freezer	−18	−18	6 months	untreated		ABTS							increase	fw	[16]
kale	blast freezer	−20	−20	12 months	blanching		ABTS					no effect	no effect		fw	[98]
	blast freezer	−30	−30	12 months	blanching							no effect	no effect		fw	[98]
	nr	−22			blanching		ABTS					no effect			fw	[110]
onion	flo-freezer	nr	ns		blanching					ORAC		increase			fw	[105]
peas	fluidization tunnel	−18	−18	12 months	blanching		ABTS	DPPH			Fe^2+^			DRM	dw	[118]
	nr	−40	−20	6, 12, 18 moths	blanching			DPPH					decrease		dw	[103]
	nr	−30	−20	21 days	blanching				FRAP			decrease	no effect		fw	[102]
	home freezer	−18	−18	6 months	untreated		ABTS							increase	fw	[16]
potato	nr	−40	−20	6, 12, 18 months	blanching			DPPH					no effect		dw	[103]
spinach	flow-freezer	nr	ns		blanching					ORAC		decrease			fw	[105]
	nr	−40	−20	6, 12, 18 months	blanching			DPPH					no effect		dw	[103]
	industrial freezing	nr			blanching					ORAC		decrease			dw	[106]
	nr	−30	−20	21 days	blanching				FRAP			decrease	no effect		fw	[102]
string beans	fluidization tunnel	−18	−18	12 months	blanching		ABTS	DPPH			Fe^2+^			DRM	dw	[118]
swede	nr	−40	−20	6, 12, 18 months	blanching			DPPH					slight decrease		dw	[103]
tomato	home freezer	−18	−18	6 months	untreated		ABTS							no effect	fw	[16]
turnip greens	chamber freezer	−30	−30	up to 28 days	untreated			DPPH						decrease	dw	[119]
	chamber freezer	−30	−30	up to 28 days	blanching			DPPH						decrease	dw	[119]
yellow pepper	home freezer	−18	−18	6 months	untreated		ABTS							decrease	fw	[16]
zucchini	home freezer	−18	−18	6 months	untreated		ABTS							increase	fw	[16]

ORAC: Oxygen Radical Absorbance Capacity; FRAP: Ferric Reducing Antioxidant Power; ABTS: 2,2′-azinobis (3-ethylbenzothiazoline-6-sulfonic acid) radical cation assay; DPPH: 2,2-diphenyl-1-picrylhydrazyl free radical assay; β-CB: β-carotene bleaching; Fe^2+^: Fe^2+^ chelating ability; IQF: Individual Quick Freezing; dw: dry weight; fw: fresh weight; nr: not reported; ns: no storage; DRM: discordant results between the method applied for the antioxidant activity (AOA) determination; DRP: different results depending on the blanching pre-treatment applied.

**Table 2:** Single and combined effect of freezing and frozen storage on the antioxidant activity (AOA) of fruits.

												Effects				
Fruit	Product Type (Whole, Puree, Juices)	Freezing System	Freezing T (°C)	Storage T (°C)	Storage Times	Pre-Treatment	AOA Test					Freezing	Storage	Freezing and Frozen Storage	Data Expression	References
acerola	puree	not reported	−18	−18	3 months	untreated	ABTS	DPPH						decrease	fw	[137]
apple	juice	home freezer	−18	−18	10 days	untreated		DPPH					no effect		fw	[133]
apple	juice	liquid nitrogen	−70	−70	10 days	untreated		DPPH					no effect		fw	[133]
apple conventional cv. Golden Delicious	whole	blast freezer	−40	−40	10 months	untreated	ABTS							decrease	dw	[73]
apple cv. Fuji	puree	lab freezer	−18	−18	1, 6 months	untreated	ABTS	DPPH				decrease	DRM		fw	[130]
apple cv. Gold Rush	puree	lab freezer	−18			untreated	ABTS					decrease			fw	[131]
apple cv. Granny Smith	puree	lab freezer	−18			untreated	ABTS					decrease			fw	[131]
apple cv. Idared	puree	lab freezer	−18	−18	1, 6 months	untreated	ABTS	DPPH				decrease	DRM		fw	[130]
apple organic cv. Golden Delicious	whole	blast freezer	−40	−40	10 months	untreated	ABTS							decrease	dw	[73]
blackberry	whole	liquid nitrogen cabinet	−80	−24	0, 3, 6, 9 months	untreated		DPPH				decrease	no effect		fw	[127]
blackberry	whole	IQF	nr	−18	2, 4, 6, 8, 10 months	untreated			FRAP			no effect	decrease		fw	[122]
blackberry cv. Cacanska bestrna	whole	not reported	−18	−18	5 weeks	untreated		DPPH						decrease	fw	[135]
blackberry cv. hornfree	whole	not reported	−18	−18	5 weeks	untreated		DPPH						decrease	fw	[135]
blueberry	whole	IQF	nr	−18	2, 4, 6, 8, 10 months	untreated			FRAP			no effect	decrease		fw	[122]
blueberry	whole	home freezer	−18	−18	1, 3, 6 months	untreated	ABTS	DPPH						increase	fw	[48]
blueberry	whole	not reported	−20	−20	1, 3 months	untreated		DPPH						no effect	fw	[134]
blueberry	whole	home freezer	−18	−18	0, 2, 4, 6 months	untreated	ABTS					no effect	no effect		fw	[123]
blueberry	whole	home freezer	−35	−35	0, 2, 4, 6 months	untreated	ABTS					no effect	no effect		fw	[123]
cashew	puree	not reported	−18	−18	3 months	untreated	ABTS	DPPH						DRM	fw	[137]
cherry	whole	liquid nitrogen		−23	3, 6 months	untreated			FRAP	ORAC				decrease	fw	[51]
cherry	whole	liquid nitrogen		−70	3, 6 months	untreated			FRAP	ORAC				increase	fw	[51]
dates cv. Khalas	whole	not reported	−20	−20	1 month	untreated			DPPH	FRAP	DR			DRM	dw	[148]
dates cv. Khunaizi	whole	not reported	−20	−20	1 month	untreated			DPPH	FRAP	DR			DRM	dw	[148]
guava	puree	lab freezer	−20	−20	0, 1, 2, 3 months	untreated			FRAP	ORAC		decrease	DRM		dw	[125]
guava	puree	not reported	−18	−18	3 months	untreated	ABTS	DPPH						decrease	fw	[137]
haskap berries cv. berry blue	whole	not reported	−18	−18	1, 2, 3, 4, 5, 6 months	blanching		DPPH						decrease	fw	[99]
haskap berries cv. berry blue	whole	not reported	−18	−18	1, 2, 3, 4, 5, 6 months	untreated		DPPH						decrease	fw	[99]
haskap berries cv. berry blue	whole	not reported	−32	−32	1, 2, 3, 4, 5, 6 months	untreated		DPPH						decrease	fw	[99]
haskap berries cv. Indigo Gem	whole	not reported	−18	−18	1, 2, 3, 4, 5, 6 months	blanching		DPPH						decrease	fw	[99]
haskap berries cv. Indigo Gem	whole	not reported	−18	−18	1, 2, 3, 4, 5, 6 months	untreated		DPPH						decrease	fw	[99]
haskap berries cv. Indigo Gem	whole	not reported	−32	−32	1, 2, 3, 4, 5, 6 months	untreated		DPPH						decrease	fw	[99]
haskap berries cv. tundra	whole	not reported	−18	−18	1, 2, 3, 4, 5, 6 months	blanching		DPPH						decrease	fw	[99]
haskap berries cv. tundra	whole	not reported	−18	−18	1, 2, 3, 4, 5, 6 months	untreated		DPPH						decrease	fw	[99]
haskap berries cv. tundra	whole	not reported	−32	−32	1, 2, 3, 4, 5, 6 months	untreated		DPPH						decrease	fw	[99]
makiang	whole	lab freezer	−20	−20	0, 1, 2, 3 months	untreated			FRAP	ORAC		no effect	DRM		dw	[125]
maluod	whole	lab freezer	−20	−20	0, 1, 2, 3 months	untreated			FRAP	ORAC		no effect	decrease		dw	[125]
nectarine	whole	freeze chamber	−79	−18	1 month	untreated	ABTS							decrease	fw	[136]
orange	juice	tunnel	−40	−40	1 month	pasteurization		DPPH						no effect	fw	[149]
orange	juice	home freezer	−18	−18	10 days	untreated		DPPH					no effect		fw	[133]
orange	juice	liquid nitrogen	−70	−70	10 days	untreated		DPPH					no effect		fw	[133]
pomegranate	juice	home freezer	−25	−25	5, 10, 15, 20 days	untreated		DPPH						decrease	fw	[138]
raspberry	whole	IQF	nr	−18	2, 4, 6, 8, 10 months	untreated			FRAP			no effect	decrease		fw	[122]
raspberry	whole	commercial plant	−18	−18	12 months	untreated	ABTS							no effect	fw	[30]
raspberry cv rubis	whole	liquid nitrogen cabinet	−80	−24	0, 3, 6, 9 months	untreated		DPPH				decrease	no effect		fw	[127]
raspberry cv zeva	whole	liquid nitrogen cabinet	−80	−24	0, 3, 6, 9 months	untreated		DPPH				decrease	no effect			[127]
Raspberry cv. Autumn bliss	whole	liquid nitrogen cabinet	−80	−24	0, 3, 6, 9 months	untreated		DPPH				no effect	no effect			[127]
raspberry cv. heritage	whole	liquid nitrogen cabinet	−80	−24	0, 3, 6, 9 months	untreated		DPPH				no effect	no effect		fw	[127]
serviceberry	whole	not reported	−20	−20	10 months	untreated		DPPH	FRAP			no effect	DRM		fw	[124]
strawberry	whole	home freezer	−23	−27	13 weeks	untreated		DPPH						no effect	fw	[48]
strawberry	whole	home freezer	−27	−27	13 weeks	untreated		DPPH						no effect	fw	[48]
strawberry	puree	forced air	−25	−18	6 months	pasteurization	ABTS	DPPH				decrease	decrease		fw	[129]
strawberry	puree	forced air	−25	−18	6 months	untreated	ABTS	DPPH				decrease	increase		fw	[129]
strawberry	puree	static air	−18	−18	6 months	pasteurization	ABTS	DPPH				decrease	decrease		fw	[129]
strawberry	puree	static air	−18	−18	6 months	untreated	ABTS	DPPH				decrease	no effect		fw	[129]
strawberry	whole	liquid nitrogen		−18	up to 12 months	untreated	ABTS					no effect	increase		dw	[31]
strawberry	whole	liquid nitrogen		−80	up to 12 months	untreated	ABTS					no effect	decrease		dw	[31]
strawberry	puree	not reported	−18	−18	3 months	untreated	ABTS	DPPH						DRM	fw	[137]
strawberry	whole	home freezer	−30			untreated	ABTS					decrease			dw	[128]

ORAC: Oxygen Radical Absorbance Capacity; FRAP: Ferric Reducing Antioxidant Power; ABTS: 2,2′-azinobis (3-ethylbenzothiazoline-6-sulfonic acid) radical cation assay; DPPH: 2,2-diphenyl-1-picrylhydrazyl free radical assay; DR: deoxyribose degradation assay. IQF: Individual Quick Freezing; dw: dry weight; fw: fresh weight; nr: not reported; DRM: discordant results between the method applied for the antioxidant activity (AOA) determination; DRP: different results depending on the blanching pre-treatment applied.

**Table 3:** Evaluation of antioxidant activity (AOA) of fruits and vegetables subject to freezing pre-treated with cryoprotectants.

Food	Product Type (Whole, Puree)	Freezing System	Freezing T (°C)	Storage T (°C)	Storage Times	Cryoprotectant	AOA Test			Effect on Antioxidant Biomolecules	References
apple	puree	lab freezer	−18	−18	6 months	Sucrose, Glucose, Fructose, Trehalose (1%, 5%; *w*/*w*)	ABTS	DPPH		Positive effect on phenolic profile of Fuji variety by Sucrose (5%), Trehalose (5%) and Glucose (5%) during frozen storage. Contradictory results for Idared variety phenolic profile	[130]
	puree	home freezer	−18		ns	Sucrose, Glucose, Fructose, Trehalose (1%, 5%; *w*/*w*)	ABTS			Higher polyphenols retention observed in freeze-dried samples added with Fructose (1%) and Trehalose (1%). Higher polyphenols retention observed in frozen samples added with Sucrose (5%) and Trehalose (5%)	[131]
arazá	puree	nr	−20	−20	1, 2, 3, 4 months	Sucrose (20%; *w*/*w*)	ABTS	DPPH	FRAP	Sample added with Sucrose (20%) showed higher total phenolic content and vitamin C concentration during frozen storage	[187]
blackberries	whole	nr	−18	−18	11 months	Sucrose, Fructose, Glucose, Fructose:Glucose, Fructose:Sucrose (50%; *w*/*w*)		DPPH		Fructose and Glucose had a positive impact on total phenolic content and anthocyanins retention. Discordant effects observed on vitamin C retention	[135]
(sour) cherry	puree	nr	−18	Room temperature after freeze-drying	30 days	Maltose, Sucrose, Trehalose (5%, 10%, 20%; *w*/*w*)	ABTS	DPPH	FRAP	Negative effect of sugars on total phenolic and flavonoids content. Discordant results on single anthocyanins retention, Maltose (20%) was the only sugar showing a positive effect on anthocyanins preservation	[186]
cherry	whole	Air blast freezer	−50	−20	ns	Glucose:Fructose (2:1 ratio, solution 60%; *w*/*w*)		DPPH		-	[185]
	whole	nr	−20	−20	31 days	Antifreeze proteins		DPPH		Addition of ice structuring proteins better preserved total phenolic and anthocyanins content	[178]

FRAP: Ferric Reducing Antioxidant Power; ABTS: 2,2′-azinobis (3-ethylbenzothiazoline-6-sulfonic acid) radical cation assay; DPPH: 2,2-diphenyl-1-picrylhydrazyl free radical assay; nr: not reported; ns: no storage.
